# CCR8 leads to eosinophil migration and regulates neutrophil migration in murine allergic enteritis

**DOI:** 10.1038/s41598-019-45653-7

**Published:** 2019-07-03

**Authors:** Frank Blanco-Pérez, Yoichiro Kato, Irene Gonzalez-Menendez, Jonathan Laiño, Masaharu Ohbayashi, Manja Burggraf, Maren Krause, Jörg Kirberg, Yoichiro Iwakura, Manuela Martella, Leticia Quintanilla-Martinez, Noriyuki Shibata, Stefan Vieths, Stephan Scheurer, Masako Toda

**Affiliations:** 1 Vice President Research Group “Molecular Allergology”, Paul-Ehrlich-Institut, Langen, Germany; 2 Department of Pathology, Tokyo Women’s Medical University, Tokyo, Japan; 3 Institute of Pathology and Neuropathology, Eberhard Karls University of Tübingen and Comprehensive Cancer Center, University Hospital Tübingen, Tübingen, Germany; 4 Department of Nursing, Graduate School of Health Sciences, Toyohashi SOZO University, Toyohashi, Japan; 5 Junior Research Group 1 Experimental Allergy Models”, Paul-Ehrlich-Institut, Langen, Germany; 6 Division of Immunology, Paul-Ehrlich-Institut, Langen, Germany; 7 Center for Animal Disease Models, Research Institute for Biomedical Sciences (RIBS), Tokyo University of Science (TUS), Chiba, Japan; 8 Laboratory of Food and Biomolecular Science, Graduate School of Agricultural Science, Tohoku University, Sendai, Japan

**Keywords:** Acute inflammation, Inflammation

## Abstract

Allergic enteritis (AE) is a gastrointestinal form of food allergy. This study aimed to elucidate cellular and molecular mechanisms of AE using a murine model. To induce AE, BALB/c wild type (WT) mice received intraperitoneal sensitization with ovalbumin (an egg white allergen) plus ALUM and feeding an egg white (EW) diet. Microarray analysis showed enhanced gene expression of CC chemokine receptor (CCR) 8 and its ligand, chemokine CC motif ligand (CCL) 1 in the inflamed jejunum. Histological and FACS analysis showed that CCR8 knock out (KO) mice exhibited slightly less inflammatory features, reduced eosinophil accumulation but accelerated neutrophil accumulation in the jejunums, when compared to WT mice. The concentrations of an eosinophil chemoattractant CCL11 (eotaxin-1), but not of IL-5, were reduced in intestinal homogenates of CCR8KO mice, suggesting an indirect involvement of CCR8 in eosinophil accumulation in AE sites by inducing CCL11 expression. The potential of CCR8 antagonists to treat allergic asthma has been discussed. However, our results suggest that CCR8 blockade may promote neutrophil accumulation in the inflamed intestinal tissues, and not be a suitable therapeutic target for AE, despite the potential to reduce eosinophil accumulation. This study advances our knowledge to establish effective anti-inflammatory strategies in AE treatment.

## Introduction

The prevalence of food allergy appears to have increased over the past decade. Food allergy causes clinical symptoms systemically and/or locally in cutaneous, respiratory, ocular, and gastrointestinal tissues^1–3^. Gastrointestinal forms of food allergy, which include allergic enteritis and colitis, are observed most frequently in pediatric patients with cow’s milk, or soy allergy, and also in adult patients allergic to other foods, including eggs of hens and wheat^4,5^. Biopsies have shown varying degrees of villous atrophy, tissue edema, crypt abscess formation, and eosinophil infiltration in the inflamed tissues of patients with allergic enteritis and colitis^6^. In addition to eosinophils, infiltration of lymphocytes, neutrophils, and/or mast cells has also been observed^6,7^. However, compared to other clinical phenotypes of food allergy, such as atopic dermatitis, allergic asthma, urticaria and anaphylaxis, the pathological mechanism underlying allergic enteritis is not well understood.

We previously established a murine model of allergic enteritis (AE) using ovalbumin (OVA, a major allergen of egg white) as model allergen^8^. To this end, BALB/c mice were sensitized intraperitoneally (i.p.) by injection with OVA in alum and challenged by intake of an egg white (EW) diet. The OVA-sensitized mice on the EW-diet exhibited clinical symptoms, e.g., weight loss, decrease in body temperature, and inflammation in the small intestine^8,9^. To elucidate the molecular and cellular mechanism underlying AE, in this study, we performed a microarray gene analysis using the described model. Microarray analysis showed that, among many others, the gene expression of CC chemokine receptor 8 (CCR8) and its ligands, CC chemokine ligand 1 (CCL1, known as I309 in human and TCA3 in mice) was upregulated in inflamed intestinal tissues of AE mice.

Leukocyte trafficking is controlled by tissue-specific expression of chemokines and chemokine receptor expression on the cell surface of leukocytes^10,11^. CCR8 expression has been detected in various types of immune cells, including diverse subsets of T cells (Th1 cells, Th2 cells, and T-reg cells), monocytes, dendritic cells (DCs), macrophages and epithelial cells, in a tissue and inflammatory status-dependent manner^12–17^. Several studies showed an essential role of the CCR8/CCL1 axis in the recruitment of Th2 cells and the development of inflammation in murine models of allergic asthma or atopic dermatitis^18–24^. Based on these studies, the potential of CCR8 antagonists to treat allergic asthma has been discussed^25,26^. However, the importance of CCR8 in allergic asthma has been debated, since additional studies have shown a dispensable role of CCR8 in the recruitment of Th2 cells into inflamed respiratory tissues of experimental mice^27–29^.

CCR8 also induced homing of skin IL-10 producing T cells to the inflamed tissue^30^. Yabe *et al*. showed that CCR8 regulates migration of DCs from the skin to the draining lymph nodes in inflammation related to contact allergy (Type IV allergy)^31^. The role of CCR8 in the development of AE remains to be elucidated. In the present study, we aimed to assess the role of CCR8 in the development of AE using CCR8KO mice. We found that the absence of CCR8 engagement reduces expression of CCL11 (eotaxin-1) and eosinophil accumulation but enhances neutrophil accumulation in the AE tissues.

## Materials and Methods

### Animals

BALB/c mice and CCR8KO mice (C.129P2-Ccr8^tm1Yiw^) on BALB/c N8 background^31^ were bred and maintained under pathogen free conditions in the animal facility of the Paul-Ehrlich-Institut. Animal experiments were performed in compliance with national law approved by local authority: Regierungspräsidium Darmstadt (the Darmstadt Regional Administrative Council, Germany). License number is F107/1020.

### Induction of AE

Mice (female, 6 to 8 weeks old) were sensitized with 10 μg of OVA (grade V, Merck KGaA, Darmstadt, Germany) and 1 mg of ALUM (Thermo Fisher Scientific, Darmstadt, Germany) in 500 µl of PBS, or treated only with PBS or Alum by i.p. injection twice at a two-week interval^8^. Two weeks after the second sensitization, the mice were fed an EW-diet for 7 days as the longest. The EW diet is a pellet-based diet containing 100% EW as source of 20% of the protein^8^. The diets were prepared at ssniff Spezialdiäten GmbH (Soest, Germany).

### Microarray analysis

**Figure 1:**
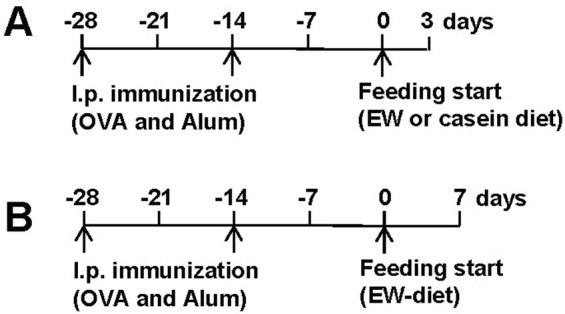
Immunization schedule. BALB/c mice were i.e. sensitized with OVA plus ALUM twice at a two-week’s interval. (**A**) In order to perform microarray analysis, two weeks after the last sensitization, the mice were fed EW-diet or casein diet for three days. (**B**) In the majority of experiments, two weeks after the last sensitization, the mice were fed EW diet for seven days.

BALB/c mice were sensitized with OVA plus ALUM twice at two-week intervals and fed EW-diet, or a control casein (CN) diet containing a cow CN as source of 20% of the protein (ssniff Spezialdiäten GmbH) for 3 days (see immunization schedule in Fig. 1A). The jejuna were harvested from the mice and homogenized in Trizol reagent (Thermo Fisher Scientific, Bonn, Germany) for RNA extraction. RNA amplification, labelling, cRNA microarray hybridization, gene expression analyses and bioinformatics analyses were performed at Miltenyi Biotec Genomic Services (Bergisch Galdbach, Germany). To produce Cy3-labeled cDNA, RNA samples were amplified and labeled using the Agilent Low Input Quick Amp Labelling kit (Agilent Technologies, California, USA). The Cy3-labeled fragmented cDNA (1.65 µg) was hybridized overnight (17 hours, 65 °C) to an Agilent Whole Mouse Genome Oligo Microarrays 4 × 44 K using the Agilent Gene Expression Hybridization kit (Agilent Technologies). Finally, the microarrays were washed, and fluorescence signals of the hybridized Agilent Microarrays were detected using Agilent’s Microarray Scanner System. The Agilent Feature Extraction Software (FES) was used to read out and process the microarray image files. For determination of differential gene expression, FES derived output data files were analyzed using the Rosetta Resolver gene expression data analysis system (Rosetta Biosoftware, Washington DC, USA).

In bioinformatics analyses, the intensity data was background corrected and quantile normalization was conducted between the arrays. The normalized intensities were log2 transformed and used as a basis for further analysis. Significant expression differences between sample groups were determined by a two-sided t-test with equal variance on the normalized log2 intensity data. The statistical tests were complemented by a non-statistical quantification of the mean expression difference between the conditions. The mean distances between the sample groups were computed (the mean log2 normalized intensity data for each group was subtracted from one another). The fold change and log2-ration values between the samples were appended to the t-test results. Additional to an adjusted p-value ≤ 0.05, genes selected as reliable candidates were required to show an at least 2-fold average expression difference between the groups.

### Measuring serum levels of antibodies

The serum levels of OVA-specific IgE, IgG1 and IgG2a Abs were measured by ELISA as reported previously^8^. Briefly, ELISA antibodies are listed in Table S3. Microtiter plates (Sarstedt, Nümbrecht, Germany) were coated with 100 µg/ml of OVA in 50 mM sodium carbonate buffer (pH 9.6) at 4 °C overnight. After blocking with 5% BSA, serum samples were applied to the wells and IgE binding was detected by biotin-conjugated rat anti-mouse IgE antibodies (R35–72, BD Bioscience, Heidelberg, Germany) and HRPO-conjugated streptavidin (BD Bioscience). For detection of IgG1 and IgG2a antibodies, HRPO-conjugated goat anti-mouse IgG1 and HRPO-conjugated rabbit anti-mouse IgG2a antibodies were used (Thermo Fisher Scientific). The peroxidase substrate was 3, 3′, 5, 5′-tetramethylbenzidine (BD Bioscience).

### Measuring T-cell cytokine production

Mesenteric lymph nodes (MLN) and spleens were isolated from OVA-sensitized mice on day 7 of EW-diet. CD4^+^ T-cells were isolated from MLNs and spleen using an isolation kit (Miltenyi Biotec). CD4^+^ T-cells (1.0 × 10^6^ cells/ml) and mitomycin-treated syngenic splenocytes (2.0 × 10^6^ cells/ml) were stimulated with 1.0 mg/ml of LPS free OVA for 72 h. The concentrations of IL-4 and IL-5 in the culture supernatant were determined using ELISA. Antibodies used in cytokine ELISA are listed in Table S3.

### Measuring T-cell frequency in spleens and MLNs

MLNs and spleens were isolated from OVA-sensitized and/or EW-diet-fed mice. CD4^+^ T-cells in the tissue’s cell suspension were stained with PE-conjugated anti-mouse CD4 mAb, APC-conjugated anti-mouse CD25 mAb and PE-Cy5-conjugated anti-mouse Foxp3 mAb after FcR-blocking using anti-CD16/CD32 monoclonal antibody (Thermo Fisher Scientific). Cell permeabilization for Foxp3 staining was performed using the Anti-mouse/Rat Foxp3 Staining set (Thermo Fisher Scientific). Data was analyzed using LSR II flow cytometer and FlowJo Engine v3.04910 (BD Bioscience). Antibodies used in FACS analysis are listed in Table S4.

### Analysis of eosinophil and neutrophil frequency in intestinal lamina propria cells

Intestinal lamina propria cells were prepared according to a protocol described by Weigmann *et al*. with slight modifications^32^. BALB/c mice were sensitized with OVA plus ALUM twice at two-week intervals and fed EW-diet for 3 days (see immunization schedule in Fig. 1B). Small intestines were harvested from OVA-sensitized, or non-sensitized mice on day 7 of EW-diet. After removal of Peyer´s patches, intestines were cut into 4–5 cm pieces, wash with cold PBS, and opened longitudinally. The tissues were then cut into 1 cm. pieces, and treated with HBSS (Thermo Fisher Scientific) containing 5 mM DTT (Molekula, Dorset, UK) at 37 °C for 20 min, and HBSS containing 5 mM EDTA and 10 mM HEPES at 37 °C for 20 min. Remaining pieces were washed with HBSS containing 10 mM HEPES at 37 °C for 10 min, and digested in PBS containing 500 µg/ml Collagenase D (Merck KGaA, Darmstadt, Germany), 500 µg/ml DNase I (Merck KGaA) and 0.5 U/ml Dispase II (Merck KGaA). After washing with PBS, the cells were treated with anti-CD16/CD32 mAb, Fixable Viability Dye eFluor 450 (Thermo Fisher Scientific), and stained with FITC-conjugated anti-CD45 mAb and eFluor 660-conjugated anti-CD170 (SiglecF) mAb to identify eosinophils, or with FITC-conjugated anti-CD45 mAb, PE-Cy5-conjugated anti-CD11b mAb and PE-conjugated anti-Ly6G mAb to identify neutrophils. Antibodies used in FACS are listed in Table S4.

### Measuring cytokine, chemokine and mMCP1 concentrations in intestinal tissue homogenates

Longitudinal sections of intestinal tissue (5 cm) were taken from the jejunum as described above. Peyer’s patches were removed. The tissue was washed with cold PBS and frozen in liquid nitrogen. Tissues were minced using a mortar and pistil until obtain a fine powder. The powder was resuspended in 300 µl of cold PBS containing protease inhibitors, centrifuged at 12.000 g for 20 min, and the supernatant transferred into a fresh tube. Protein concentration was determined by BCA assay (Thermo Fisher Scientific) and adjusted to 5.0 mg/ml. Concentrations of CCL1, CCL11, CCL8, IL-5, IL-13, IL-33 and mMCP1 in the homogenate were determined by ELISA. Antibodies used in the ELISA are listed in Table S3.

### Histological analysis

Longitudinal sections of intestinal tissue (2 cm) were taken from jejunum (9.5 cm distal to the duodenum). The tissues were fixed in 4% formalin and embedded in paraffin. Sections 5 µm thick were prepared and stained with hematoxylin and eosin (H&E) for morphologic analysis and detection of eosinophils. In addition, Eosinophil-Mast Cell Stain Kit (Teomics, USA) was used to visualize eosinophils.

### Statistical analysis

Comparison of mean values between different groups was performed by student t-test in GraphPad Prism 7 (San Diego, USA), or by ANOVA followed by Dunnett’s test in IBM SPSS statistics (Chicago, USA). *p* values < 0.05, and < 0.01 were designated with * and ** respectively, and considered significant.

## Results

### CCL1 expression is upregulated in the inflamed intestinal tissues of AE mice

**Figure 2:**
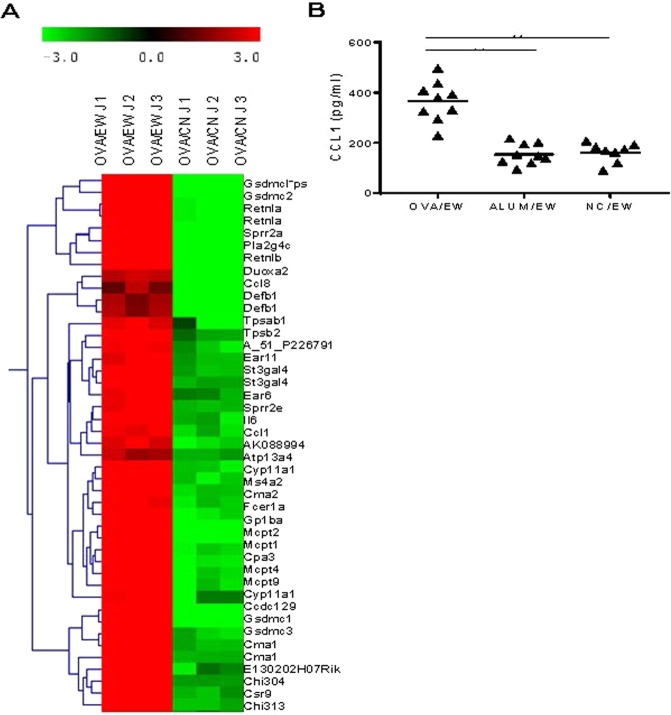
Expression of CCL1 in inflamed tissues of AE mice. (**A**) BALB/c mice (n = 3/group) were i.p. sensitized with OVA plus ALUM twice at a two-week’s interval. Two weeks after the last sensitization, the mice were fed EW-diet or casein diet for 3 days. The jejunums were harvested from the mice and the levels of gene expression in the tissues were assessed by microarray analysis. (**B**) The jejunums were harvested from mice (n = 3–4/group), which were sensitized with OVA plus ALUM, or treated with ALUM only, or non-sensitized, and fed EW-diet for 7 days. The concentrations of CCL1 in homogenates of the tissues were measured by ELISA. Each symbol represents an individual mouse. The data are pooled of two independent experiments. OVA/EW; OVA-sensitized and EW-diet fed, ALUM/EW; ALUM-treated and EW-diet fed, NC/EW; non-treated and EW-diet fed. **p < 0.01.

First, a microarray analysis on inflamed intestinal tissues of AE mice was performed in order to identify the molecules involved in the development of AE. BALB/c WT mice were i.p. sensitized with OVA in combination with ALUM, and challenged by feeding an EW-diet for 3 days to trigger AE or on a casein (CN) diet as control (see immunization schedule in Fig. 1A). Microarray analysis showed that the gene expression of CCL1 and CCL8 was highly up-regulated in the jejunum of OVA-sensitized and EW-diet fed (OVA/EW) WT mice (41.66 and 47.91 folds respectively) when compared to that in the OVA-sensitized and casein-diet fed mice, i.e., the control group (OVA/CN WT mice; Fig. 2A and Table S1).

CCL1 binds to CCR8 specifically, whereas CCL8 binds to CCR4 and CCR5 in addition with CCR8^10,11^. The enhanced gene expression of CCR8 and the presence of CCR8 expressing cells were detected in the jejunum of OVA/EW WT mice (Table S2 and Fig. S1). To verify the results of the microarray analysis, the protein concentration of CCL1 was measured in intestinal homogenates of OVA/EW WT mice, ALUM/EW mice, or non-sensitized and EW-diet-fed (NC/EW) WT mice. Increased concentrations of CCL1 in OVA/EW WT mice were then detected, when compared to those in NC/EW WT mice or OVA/CN WT mice (Fig. 2B).

### CCR8 deficiency reduced eosinophil accumulation, but enhanced neutrophil accumulation in AE tissues

To determine if CCR8 is involved in the development of clinical symptoms and AE, we used CCR8KO mice. WT and CCR8KO mice were sensitized with OVA in combination with ALUM and fed on an EW-diet for 7 days (see immunization schedule in Fig. 1B). During the EW diet, OVA/EW WT mice significantly reduced body weight and temperature, when compared to NC/EW mice (Fig. S2A–C). OVA/EW CCR8KO also reduced body weight and temperature. However, the levels of reduction in OVA/EW CCR8KO tended to be lower than those of OVA/EW WT mice. The results suggest that CCR8 is partially involved in the development of clinical symptoms induced by allergenic diet.

**Figure 3:**
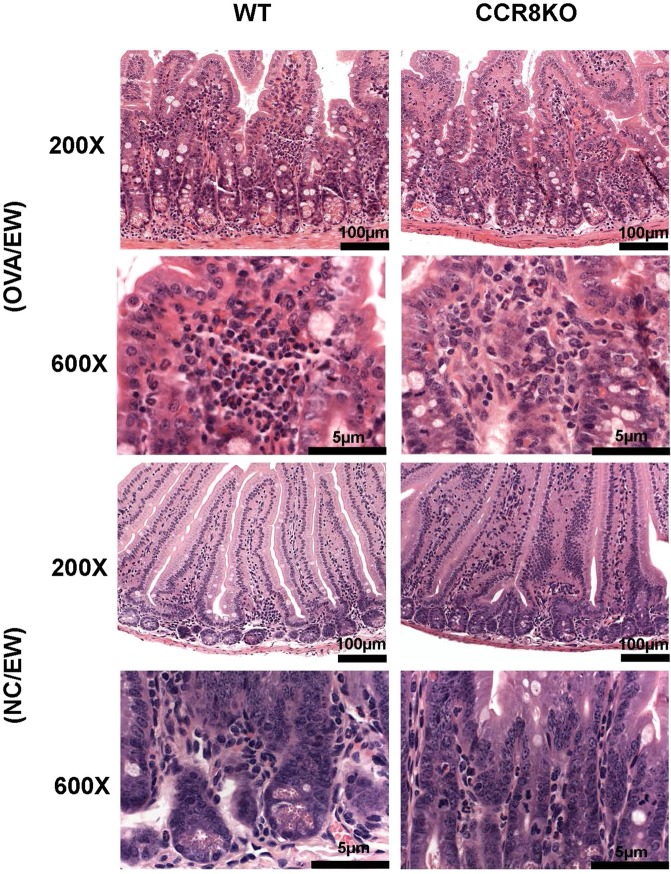
Development of AE in CCR8KO mice. WT mice and CCR8KO mice were i.p. sensitized with OVA plus ALUM, or treated only with PBS, and fed EW-diet for 7 days. The jejunums were harvested from the mice, and stained with H&E. All images were taken in same magnification. OVA/EW; OVA-sensitized and EW-diet fed, NC/EW; non-sensitized and EW-diet fed. The data are representative for three independent experiments.

**Figure 4:**
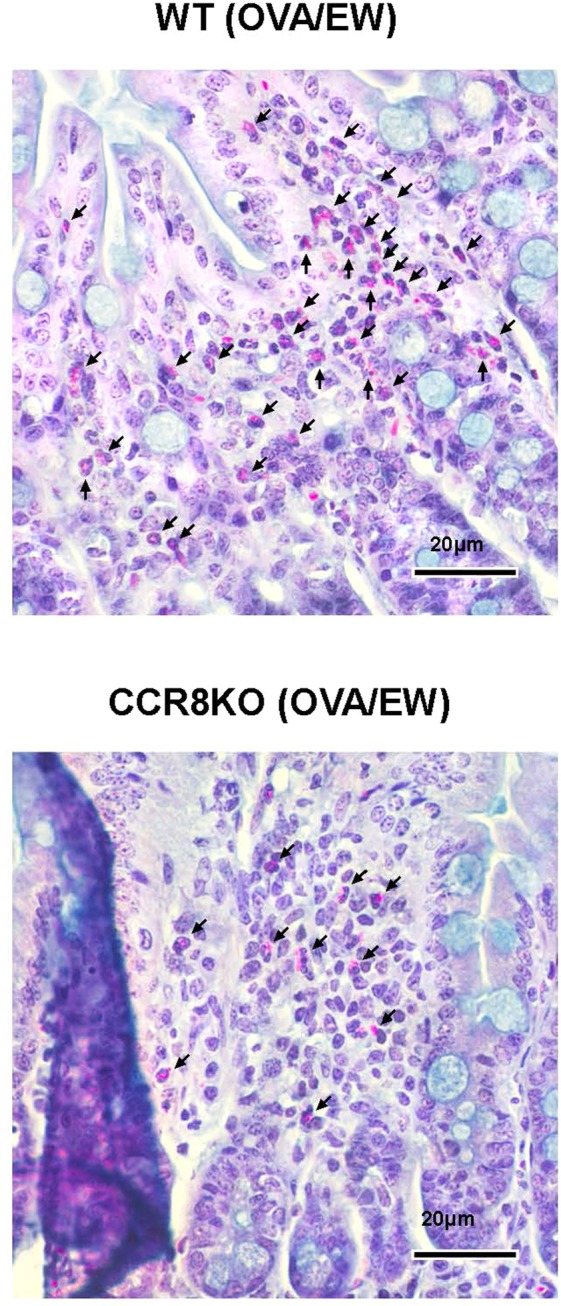
Reduced eosinophil accumulation in CCR8KO mice. WT mice and CCR8KO mice were i.p. sensitized with OVA plus ALUM, or treated only with PBS, and fed EW-diet for 7 days. (A) The harvested jejunums were stained with vital new red solution to visualize eosinophils. Arrows indicate eosinophils in the inflamed tissues of OVA/EW WT and CCR8KO mice. The data are representative for two independent experiments.

Next, we isolated jejunum tissues from the mice for histological analysis. HE-stained tissues showed that OVA/EW WT and OVA/EW CCR8KO mice developed inflammation, which is characterized by irregular villi, a thickened muscular layer, crypt elongation, and accumulation of granulocytes in the lamina propria (Fig. 3). A histology scoring analysis, which is based on grade of accumulation of granulocytes, villi morphology, and presence or absence of edema, indicated that inflammation levels were higher in OVA/EW WT mice, compared to OVA/EW CCR8KO mice (Table S5). Morphological changes were not observed in the tissues of non-sensitized mice, NC/EW WT and NC/EW CCR8KO mice (Fig. 3). Animals that received ALUM alone and were fed an EW-diet did not develop AE neither^8^. Interestingly, immunohistochemical analysis revealed that the profile of granulocyte accumulation was different in the different groups. OVA/EW WT mice presented intensive infiltration of eosinophils and neutrophils (Figs 4A and S3), while OVA/EW CCR8KO mice showed reduced accumulation of eosinophils, but increased accumulation of neutrophils (Figs 4A and S3). The numbers of mast cells were similar in both OVA/EW WT and OVA/EW CCR8KO mice (Fig. S4).

**Figure 5:**
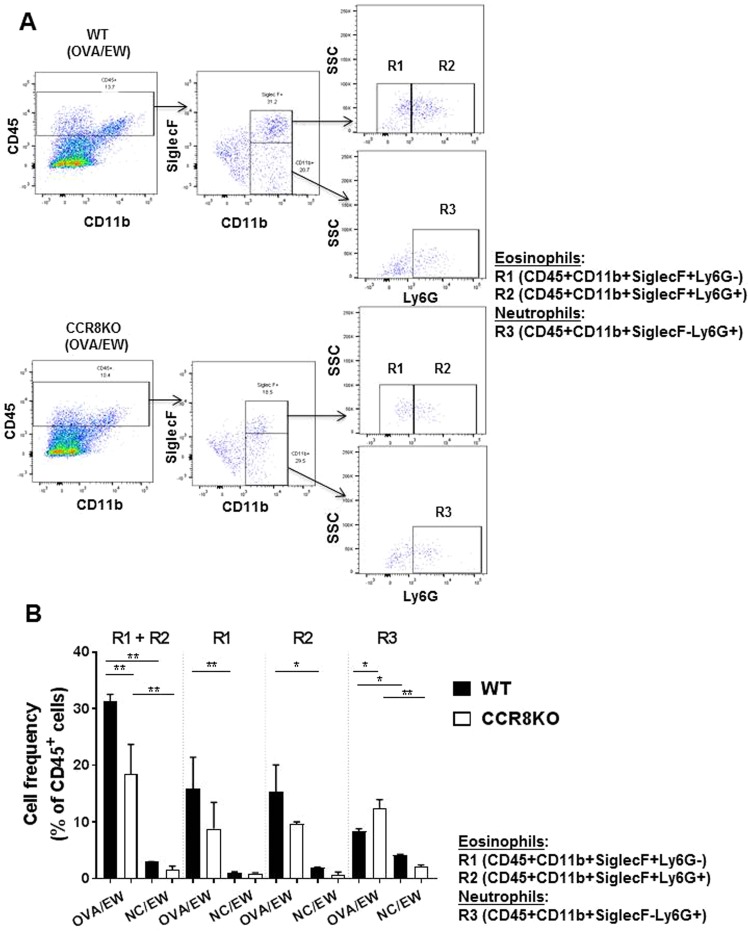
Reduced frequency of eosinophils and increased frequency of neutrophils in the inflamed intestinal tissues of CCR8KO mice. WT and CCR8KO mice (n = 4/group) were sensitized with OVA plus ALUM, and fed EW-diet for 7 days. The jejunums were harvested from the mice, and subjected to enzymatic treatment for preparation of lamina propria cells. (**A**) Eosinophils (CD11b^+^SiglecF^+^Ly6G^−^ and CD11b^+^SiglecF^+^Ly6G^+^ cells) and neutrophils (CD11b^+^SiglecF^−^Ly6G^+^ cells) in lamina propria CD45^+^ cells population were analyzed by FACS. (**B**) The frequencies of eosinophils and neutrophils in lamina propria CD45^+^ cells population were analyzed by FACS. The data in Fig. 5B are shown as mean ± standard deviation for each group (bars). All data are representative for three independent experiments. *p < 0.05, **p < 0.01.

To verify the results of the histological analysis, FACS analysis was performed using lamina propria cells; the samples were prepared using the small intestine tissues of WT or CCR8KO mice. OVA/EW WT mice and OVA/EW CCR8KO mice exhibited longer length of small intestines, compared to their NC/EW control animals (Fig. S5A). The length of intestines and the numbers of isolated lamina propria cells from OVA/EW WT mice and OVA/EW CCR8KO mice were comparable (Fig. S5A,B). However, (i) a decreased frequency of eosinophils (CD11b^+^SiglecF^+^ cells) and (ii) an increased frequency of neutrophils (CD11b^+^SiglecF^−^Ly6G^+^ cells) among the lamina propria leukocytes (CD45^+^ cells) was observed in OVA/EW CCR8KO mice, compared to that in OVA/EW WT mice (Fig. 5A,B). FACS analysis also showed presence of Ly6G^−^SSC^high^ and Ly6G^+^SSC^high^ cells in CD11b^+^SiglecF^+^ eosinophil population (Fig. 5A). A previous study reported that CD11b^+^SiglecF^+^Ly6G^+^ cells is an eosinophil subpopulation, although the cells express Ly6G, a neutrophil marker^33^. The frequencies of CD11b^+^SiglecF^+^Ly6G^−^SSC^high^ and CD11b^+^SiglecF^+^Ly6G^+^SSC^high^ cells tended to be lower in OVA/EW CCR8KO mice, compared to OVA/EW WT mice (Fig. 5B).

In addition, we tried to quantify the number of eosinophils and neutrophils in the small intestines, based on the cell frequency given by FACS analyses and the length of small intestines isolated from mice, or the numbers of lamina propria cells isolated from the small intestines. The results indicate that the numbers of eosinophils were reduced, whereas those of neutrophils were increased in OVA/EW CCR8KO mice (Fig. S5C,D). The number of leukocyte common antigen (CD45) positive cells in lamina propria cells were comparable in OVA/EW WT mice and OVA/EW CCR8KO mice (Fig. S5E). The result suggests the differential involvement of CCR8 in eosinophil and neutrophil migration to the inflamed tissues in AE.

### CCR8 deficiency partly affect Th2 immune response but no IL-5 production by T-cells

**Figure 6:**
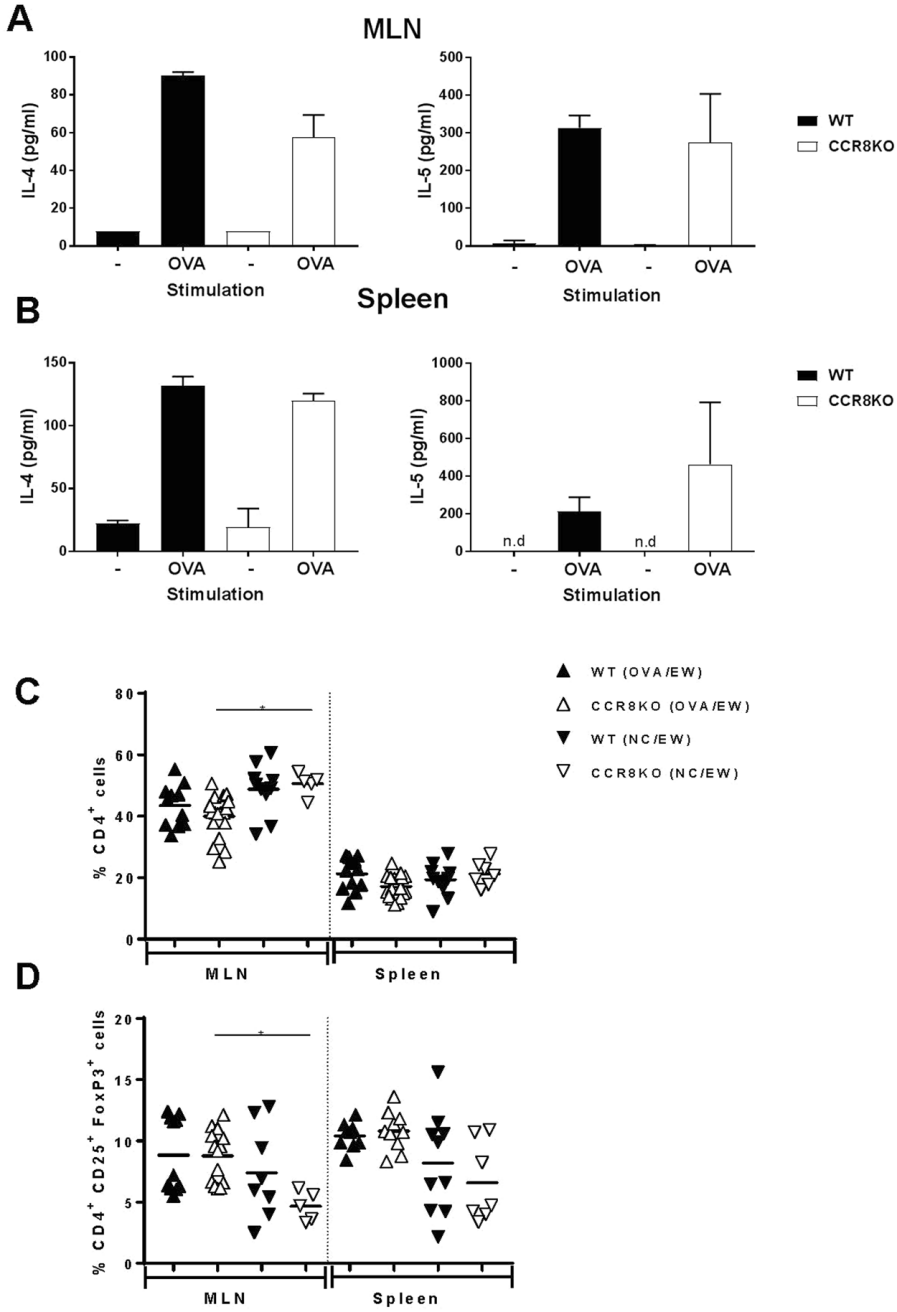
Comparable T-cell frequency and responses in WT and CCR8KO mice. WT and CCR8KO mice were sensitized with OVA plus ALUM and fed EW-diet for 7 days. CD4^+^ T-cells were isolated from the mesenteric lymph nodes (MLN) and spleen. CD4^+^ T-cells (1.0 × 10^6^ cells/ml) were co-cultured with mitomycin-treated syngenic splenocytes (2.0 × 10^6^ cells/ml) in the presence of 1 mg/ml of OVA for 72 hours. Concentrations of IL-4 and IL-5 in the cell culture supernatants of CD4^+^ T-cells isolated from (**A**) MLN, or (**B**) spleen were measured by ELISA. The data are representative for two independent experiments using n = 3/group. The data are shown as mean ± standard deviation for each group (bars). (**C**) The frequency of CD4^+^ T-cells and (**D**) the frequency of Treg (CD4^+^ CD25^+^ Fox p3^+^) cells in MLN and spleen was determined by FACS. The data are pooled of three independent experiments using n = 2–4/group. *p < 0.05.

**Figure 7:**
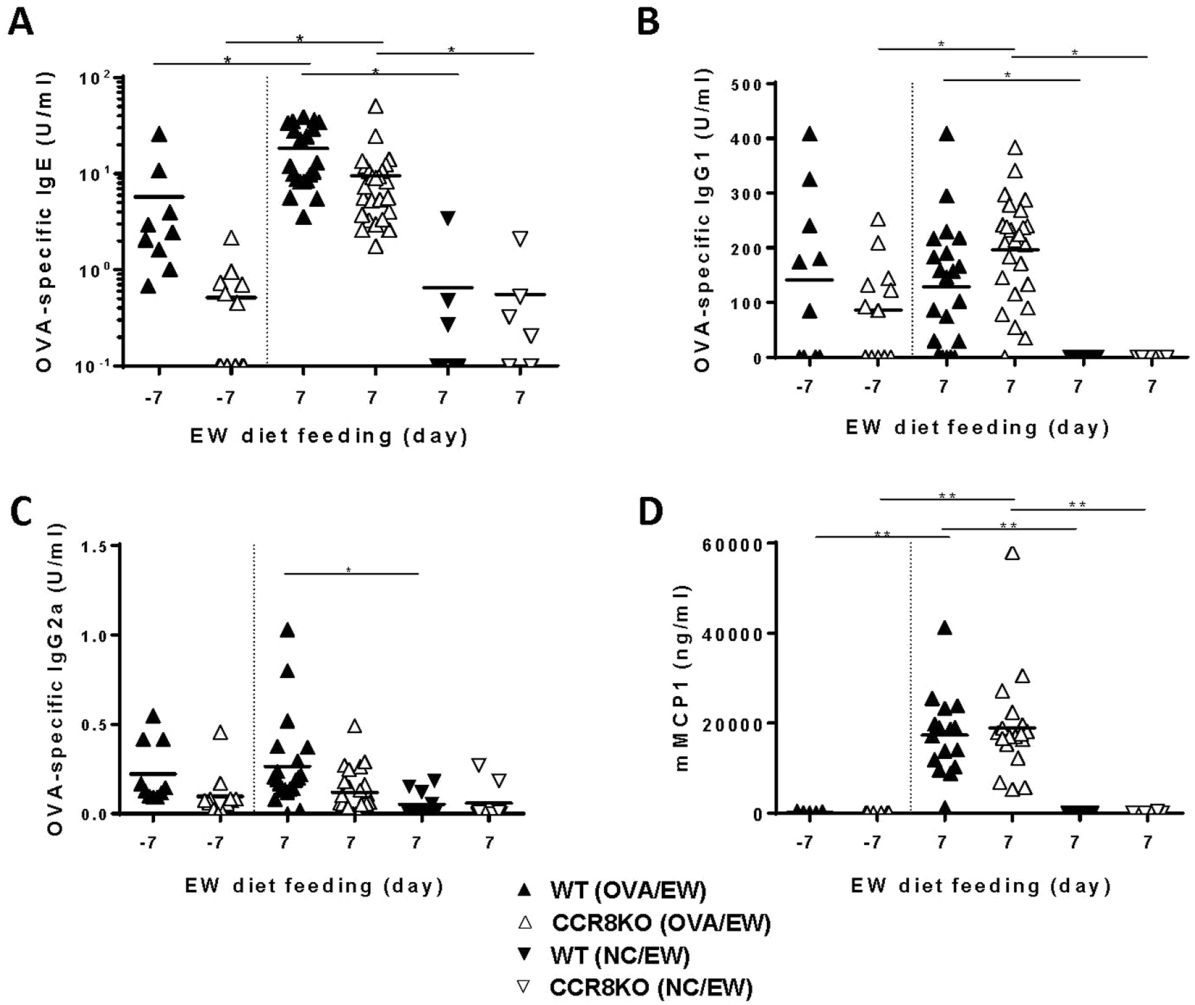
Comparable levels of OVA-specific antibody responses in WT and CCR8KO mice. WT and CCR8KO mice (n = 3–5/group) were i.p. sensitized with OVA plus ALUM, and fed EW-diet for 7 days. The serum levels of (**A**) IgE, (**B**) IgG1 and (**C**) IgG2a Abs specific for OVA and (**D**) mMCP1 on days -7 and 7 of EW-diet were measured by ELISA. Each symbol represents an individual mouse. The data are pooled of three independent experiments. *p < 0.05, **p < 0.01.

A previous study showed reduced eosinophil accumulation in allergen-induced airway inflammation of CCR8KO mice due to defective Th2 immune response^34^. To determine whether CCR8 influences the development of adaptive immunity in a murine model of AE, we assessed T-cell and antibody responses in WT and CCR8KO mice. Mesenteric lymph node (MLN)-derived T-cells and splenic T-cells from OVA/EW WT and OVA/EW CCR8KO mice produced similar levels of IL-4, and IL-5, Th2 cytokines that induce IgE production and eosinophil maturation/migration respectively in response to OVA (Fig. 6A,B). The frequencies of CD4^+^ T-cells in MLNs and spleens were comparable between OVA/EW WT and OVA/EW CCR8KO mice (p = 0.689 and p = 0.154, respectively) (Fig. 6C). There was also no significant difference in the frequency of T-reg cells (CD4^+^ CD25^+^ Fox p 3^+^ cells) in MLNs and spleens between OVA/EW WT and OVA/EW CCR8KO mice (p = 0.079 and p = 0.988, respectively), although the frequency was higher in NC/EW WT mice, compared to NC/EW CCR8KO mice (Fig. 6D). In addition, serum levels of OVA-specific IgE, IgG1, and IgG2a antibodies were comparable in both groups of mice on day 7 of EW-diet (p = 0.149, p = 0.146, and p = 0.378, respectively), although CCR8KO mice showed lower IgE and IgG2a levels before beginning the EW-diet (Fig. 7A–C). Increased levels of mMCP1, a marker of mast cells activation, were similarly detected in the sera of OVA/EW WT and OVA/EW CCR8KO mice (Fig. 7D). The results suggest that the absence of CCR8 does not influence on the development of Th2-mediated immune responses.

### CCR8 deficiency reduced CCL11 expression in AE tissues

**Figure 8:**
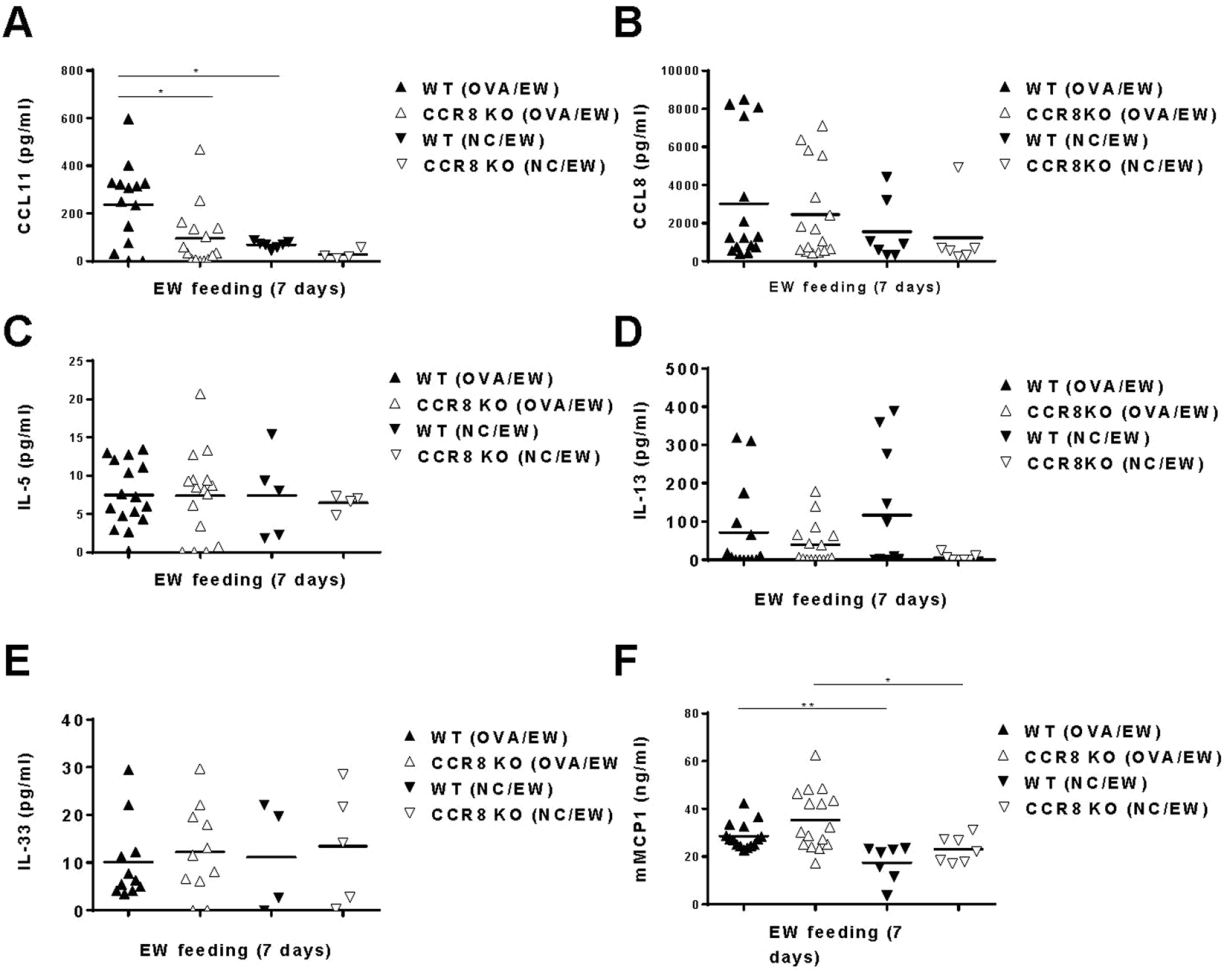
Reduced expression of CCL11 in the inflamed intestinal tissues of CCR8KO mice. WT and CCR8KO mice (n = 3–5/group) were i.p. sensitized with OVA plus ALUM, or non-sensitized, and fed EW-diet for 7 days. Small intestines were harvested from the mice. The concentrations of (**A**) CCL11, (**B**) CCL8, (**C**) IL-5, (**D**) IL-13, (**E**) IL-33 and (**F**) mMCP1 in the intestinal tissue homogenates were measured by ELISA. Each symbol represents an individual mouse. The data are pooled of three independent experiments. *p < 0.05., **p < 0.01.

In addition to IL-5, several chemokines e.g., CCL11, CCL24, and CCL26 can directly induce eosinophil migration^10,11^. Among them, enhanced gene expression of CCL11 in the intestinal tissues of OVA/EW WT mice was detected by microarray analysis (see Table S2). Therefore, we assessed whether the absence of CCR8 influences protein expression of CCL11 in the intestinal tissues of WT and CCR8KO mice. Notably, CCL11 concentrations were significantly lower in the tissue homogenates of OVA/EW CCR8KO mice, when compared to those in the tissue homogenates of OVA/EW WT mice (Fig. 8A). These results suggest that CCR8 is involved in CCL11 expression in AE sites.

In addition to the eosinophil chemoattractants, CCL8, a ligand of CCR8 potentially involved in eosinophil migration by recruiting IL-5-producing Th2 cells^35^. However, CCL8 concentrations were comparable between the tissue homogenates of OVA/EW WT and OVA/EW CCR8KO mice, suggesting that CCL8 is not crucially engaged in the eosinophil recruitment in AE (Fig. 8B).

Next, in order to analyze the expression of cytokines in the intestinal tissues, we measured Th2 type associated cytokines IL-5, IL-13, and IL-33 levels in intestinal tissues homogenates of OVA/EW WT and OVA/EW CCR8KO mice. The concentrations of IL-5 were comparable in the homogenates of OVA/EW WT, OVA/EW CCR8KO mice and their control NC/EW animals (Fig. 8C). In addition, the concentrations of IL-13 (Fig. 8D) and IL-33 (Fig. 8E) were similar in the homogenates of mice from OVA/EW WT and OVA/EW CCR8KO mice. Furthermore, the concentration of mMCP1 in the intestinal tissue homogenates (Fig. 8F) and the number of intestinal mast cells (Fig. S4) were comparable in OVA/EW WT and OVA/EW CCR8KO mice, suggesting that mast cell activation was similar in the intestinal tissues of both type of mice.

## Discussion

Eosinophils have long been observed in the inflamed tissues of allergic patients and have been proved to be therapeutic targets in allergic diseases^36,37^. Therefore, it is essential to identify which chemokine receptor and its ligands that contribute to the migration of eosinophils to allergic inflammatory sites. CCR3/CCL11 and IL-5 receptor/IL-5 are well known to act in eosinophil migration to peripheral tissues, including the gastrointestinal tract^38,39^. In this study, we have found a contribution of CCR8 in eosinophil migration to the inflamed AE tissues. However, unlike CCR3 and IL-5R, CCR8 seems to be only indirectly involved in eosinophil migration by inducing CCL11 expression. It is consistent with a previous study by Islam *et al*. showing that CCL11 expression was reduced in the skin of allergen-sensitized CCR8KO mice^35^. Furthermore, we have found that CCR8 deficiency influences neutrophil migration. CCR8KO mice showed increased neutrophil accumulation and developed AE, although eosinophil accumulation in the intestinal tissues was reduced. There is increasing evidence that neutrophils play an important role in the pathogenesis of allergic inflammation by mediating direct tissue injury or by releasing pro-inflammatory mediators^40^. Increased neutrophil numbers have also been detected in AE patients^5^. The potential of CCR8 antagonists has been considered to treat allergic asthma, since several studies have shown a role for CCR8 in the recruitment of Th2 cells and in the development of inflammation in murine models of allergic asthma^22,29,41^. However, our results suggest that CCR8 is not a suitable target in AE treatment.

Diverse roles for CCR8 in allergic asthma have been reported so far: e.g., involvement in the development of systemic Th2-type immune response and migration of Th2 cells, or regulatory T-cells, into the inflamed airway tissues^18,20,42^. Using models of *Schistosoma mansoni* soluble egg antigen-induced granuloma formation, as well as OVA and cockroach antigen-induced asthma, Chensue *et al*. have shown that eosinophil recruitment is reduced in CCR8KO mice^34^. This reduction in eosinophil recruitment in inflamed airway tissues was explained by a systemic reduction in IL-5 concentration due to defective development of Th2-type immune responses. Islam *et al*. have shown that CCR8 recruits IL-5 expressing Th2 cells in atopic dermatitis using CCR8KO mice^35^. However, we detected IL-5 production in splenic and MLN T-cells from OVA/EW WT and OVA/EW CCR8KO mice comparably. In addition, IL-5 concentrations in intestinal tissue homogenates of both mice were comparable. The frequency of regulatory T cells was comparable in spleens and MLNs of both mice. These results suggest that a defect in systemic Th2-type immune response or induction of regulatory T cells is not the main mechanism underlying reduced eosinophil accumulation in the inflamed tissues of CCR8KO mice.

Several studies have shown that histamine released from mast cells induces CCL11 expression in epithelial cells of allergen-challenged skin and lung. However, it is very unlikely that CCR8 is involved in mast cell activation and, subsequently, in CCL11 expression by epithelial cells. This postulation is supported by the fact that the number of mast cells and the concentration of mMCP1, a marker of mast cell activation, were comparable in intestinal tissues of OVA/EW WT and OVA/EW CCR8KO mice. In addition to epithelial cells, type 2 innate lymphoid cells (ILC2) could be a source of CCL11^43^. ILC2 have been associated with the allergic sensitization to foods due to their capacity to produce high amounts of IL-5 and IL-13 in intestinal mucosa^44^. IL-33 has been reported to induce activation of ILC2^44,45^. However, it is also unlikely that CCR8 influences the induction or activation of ILC2, since the concentrations of IL-5, IL-13, and IL-33, which are associated with ILC2 activation and function, were comparable in the intestinal tissue homogenates of OVA/EW WT and OVA/EW CCR8KO mice. Alternatively, intestinal macrophages could act as CCL11 producing cells. Waddell *et al*. have shown that F4/80 positive cells are the producer of CCL11 in inflamed colon using a murine model of colitis^46^. In our study, we found that OVA/EW WT mice exhibited higher numbers of macrophages (CD68 positive cells) in villi of their small intestines, compared to OVA/EW CCR8KO mice (Fig. S6). However, immunohistochemical analysis showed that the CD68 positive cells do not express CCR8 (Fig. S7), suggesting macrophages are not the producer of CCL11. CCL11 producing cells in the inflamed small intestines still need to be identified.

FACS analysis showed that the total frequencies of CD11b^+^SiglecF^+^Ly6G^−^ and CD11b^+^SiglecF^+^Ly6G^+^ cells were reduced in CCR8KO mice. Ly6G is a marker of neutrophils and used to identify the cells^47,48^. However, Percopo *et al*. have shown the CD11b^+^SiglecF^+^Ly6G^+^ cells are eosinophil subpopulation^33^. Several studies have shown that eosinophils tend to present higher side scatter (SSC: a parameter for cell density) than neutrophils^47,48^. Our study is consisted with the previous studies, as SSC of CD11b^+^SiglecF^+^Ly6G^−^ cells and CD11b^+^SiglecF^+^Ly6G^+^ cells were similar, and tended to be higher than that of CD11b^+^SiglecF^−^Ly6G^+^ cells (neutrophils) (see Fig. 5A). Further analysis is necessary whether functions (e.g. cytokine and chemokine production) of CD11b^+^SiglecF^+^Ly6G^−^ and CD11b^+^SiglecF^+^Ly6G^+^ cells are also similar in AE.

It was unexpected that CCR8 deficiency accelerated neutrophil accumulation in the intestinal tissues. In studies on the allergic asthma using CCR8KO mice, such accelerated neutrophil accumulation has not been reported. CCR8 deficiency might enhance expression of neutrophil chemoattractants, such as IL-8, leukotriene B4 (LTB4), and formyl-methionyl-leucyl-phenylalanine (fMLP), in intestinal tissues. The accelerated neutrophil accumulation may also be explained by a consequence of higher growth factor availability due to the less accumulation of eosinophils in CCR8KO mice. For instance, granulocyte-macrophage colony-stimulating factor stimulates both eosinophils and neutrophils and enhances survival of these cells^49,50^. Interestingly, Cheng *et al*. have shown that CCL11 expression counter-regulates accumulation of neutrophils in a murine model of endotoxemia^51^. It suggests that reduced CCL11 expression could lead to the enhanced neutrophil accumulation in the intestinal tissues of OVA/EW CCR8KO mice.

OVA/EW CCR8KO mice tended to exhibit lower levels of clinical symptoms, i.e. reduction of body weight and temperature, compared to OVA/EW WT mice. The development of clinical symptoms upon i.p. sensitization with OVA and feeding of the EW diet is induced by FcεRI-engaged mechanism in BALB/c mice (manuscript in preparation.). CCR8KO mice showed lower IgE levels before beginning the EW-diet, which could reduce the development of clinical symptoms.

A limitation of our study is that, we used BALB/c WT mice as controls, which were bred separately from CCR8KO mice, but not the littermate controls of the gene modified animals. We bred WT controls and CCR8KO mice in the same room. However, in addition to strain differences, breeding and maintenance in separated cages (even in the same room) could be factors altering gut microbiota composition in animals^52^. The intestinal immune system is connected with the vast diversity of microbiome present in the gut^53^. Therefore, we could not exclude a possibility that altered gut microbiota influenced on the levels of granulocyte accumulation and inflammation in CCR8KO mice.

In summary, we have identified a chemokine receptor that leads to eosinophil recruitment in AE sites. To our knowledge, this is the first study to show a contribution of CCR8 in eosinophil recruitment in intestinal tissues. However, our study also suggests a potential involvement of CCR8 in the regulation of neutrophil recruitment in AE tissues. In the future study, it would be necessary to assess whether CCR8 antagonists enhance neutrophil accumulation in AE. The findings in our study will have important implications to elucidate pathological mechanism for AE and to establish AE treatments that target chemokine receptors.

## Supplementary information

Supporting information

## Data Availability

The datasets generated during the current study are available upon reasonable request.
