# Supplementary material for: CCR8 leads to eosinophil migration and regulates neutrophil migration in murine allergic enteritis
Source: Sci Rep. 2019 Jul 3;9:9608. doi: 10.1038/s41598-019-45653-7 (PMC6610106; doi:10.1038/s41598-019-45653-7)
Supplement: Supplementary file 1 — Supporting information [file 41598_2019_45653_MOESM1_ESM.pdf]

Supporting information

CCR8 leads to eosinophil migration and regulates neutrophil migration in murine allergic enteritis

Frank Blanco-Pérez[1], Yoichiro Kato[2], Irene Gonzalez-Menendez[3], Jonathan Laiño[1], Masaharu Ohbayashi[4], Manja Burggraf[5], Maren Krause[1], Jörg Kirberg[6], Yoichiro Iwakura[7], Manuela Martella[3], Leticia Quintanilla-Martinez[3], Noriyuki Shibata[2], Stefan Vieths[1], Stephan Scheurer[1], Masako Toda[1,5]

[1]Vice President Research Group "Molecular Allergology", [5]Junior Research Group 1 "Experimental Allergy Models" and [6]Division of Immunology, Paul-Ehrlich-Institut, Langen, Germany.

[2]Department of Pathology, Tokyo Women's Medical University, Tokyo, Japan.

[3]Institute of Pathology and Neuropathology, Eberhard Karls University of Tübingen and Comprehensive Cancer Center, University Hospital Tübingen, 72076 Tübingen, Germany.

[4]Department of Nursing, Graduate School of Health Sciences, Toyohashi SOZO University, Toyohashi, Japan.

[7]Center for Animal Disease Models, Research Institute for Biomedical Sciences (RIBS), Tokyo University of Science (TUS), Chiba, Japan.

Corresponding author
Dr. Masako Toda
Current Address
Laboratory of Food and Biomolecular Science,
Graduate School of Agricultural Science,
Tohoku University, Sendai, Japan.
E-mail: masako.toda.a7@tohoku.ac.jp

Materials and Methods:

Microarray analysis

BALB/c mice were sensitized with OVA plus ALUM twice at two-week intervals and fed EW-diet for 3 days (see immunization schedule in Fig.1A). The jejunums were harvested from the mice and homogenized in Trizol (Thermo Fisher Scientific, Bonn, Germany) for RNA extraction. RNA amplification, labelling, cRNA microarray hybridization, gene expression analyses and bioinformatics analyses were performed at Miltenyi Biotec Genomic Services (Bergisch Galdbach, Germany). To produce Cy3-labeled cDNA, RNA samples were amplified and labeled using the Agilent Low Input Quick Amp Labelling kit (Agilent Technologies, California, USA). The Cy3-labeled fragmented cDNA (1.65 µg) was hybridized overnight (17 hours, 65°C) to an Agilent Whole Mouse Genome Oligo Microarrays 4 x 44K using the Agilent Gene Expression Hybridization kit (Agilent Technologies). Finally, the microarrays were washed, and fluorescence signals of the hybridized Agilent Microarrays were detected using Agilent's Microarray Scanner System. The Agilent Feature Extraction Software (FES) was used to read out and process the microarray image files. For determination of differential gene expression, FES derived output data files were analyzed using the Rosetta Resolver gene expression data analysis system (Rosetta Biosoftware, Washington DC, USA).

In bioinformatics analyses, the intensity data was background corrected and quantile normalization was conducted between the arrays. The normalized intensities were log2 transformed and used as a basis for further analysis. Significant expression differences between sample groups were determined by a two-sided t-test with equal variance on the normalized log2 intensity data. The statistical tests were complemented by a non-statistical quantification of the mean expression difference between the conditions. The mean distances between the sample groups were computed (the mean log2 normalized intensity data for each group were subtracted from one another). The fold change and log2-ration values between the samples were appended to the t-test results. Additional to an adjusted p-value ≤0.05, genes selected as reliable candidates were required to show an at least 2-fold average expression difference between the groups.

Analysis of eosinophil and neutrophil frequency in intestinal lamina propria cells

BALB/c mice were sensitized with OVA plus ALUM twice at two-week intervals and fed EW-diet for 3 days (see immunization schedule in Fig. 1B). Small intestines were harvested from OVA-sensitized, or non-sensitized mice on day 7 of EW-diet. After removal of Peyer´s patches, intestines were cut into 4-5 cm pieces, wash with cold PBS, and opened longitudinally. The tissues were then cut into 1 cm. pieces, and treated with HBSS (Thermo Fisher Scientific)

containing 5 mM DTT (Molekula, Dorset, UK) at 37°C for 20 min, and HBSS containing 5 mM EDTA and 10 mM HEPE at 37°C for 20 min. Remaining pieces were washed with HBSS containing 10 mM HEPES at 37°C for 10 min, and digested in PBS containing 500 µg/ml Collagenase D (Merck KGaA, Darmstadt, Germany), 500 µg/ml DNase I (Merck KGaA) and 0.5 U/ml Dispase II (Merck KGaA). After washing with PBS, the cells were treated with anti-CD16/CD32 mAb, Fixable Viability Dye eFluor 450 (Thermo Fisher Scientific), and stained with FITC-conjugated anti-CD45 mAb and eFluor 660-conjugated anti-CD170 (SiglecF) mAb to identify eosinophils, or with FITC-conjugated anti-CD45 mAb, PE-Cy5-conjugated anti-CD11b mAb and PE-conjugated anti-Ly6G mAb to identify neutrophils. Antibodies used in FACS are listed in Table S4.

Histological analysis

Longitudinal sections of intestinal tissue (2 cm) were taken from jejunum (9.5 cm distal to the duodenum). In order to assess development of inflammation, tissues were fixed in 4% formalin and embedded in paraffin. To observe lymphedema, the tissues were fixed in 4% paraformaldehyde, and frozen in OCT compound. Sections 5 mm thick were prepared and stained with hematoxylin and eosin (H&E) for assessment of inflammation levels, and with toluidine blue for detection of mast cells. Criteria for assessment of inflammation levels is indicated in Table S5.

To detect CCR8 and CD68, frozen tissues were blocked with goat sera, and stained with goat anti-mouse CCR8 polyclonal antibodies (Abcam, Tokyo, Japan) and rat anti-mouse CD68 monoclonal antibody (BioLegend, Tokyo, Japan). As isotype controls, normal Goat IgG antibodies (PM094 MBL) and Rat IgG2a antibodies (BioLegend) were used. After incubating with the primary antibodies or isotype controls, the tissues were treated with Alexa Fluor 647-conjugated donkey anti-goat IgG H&L Abs pre-adsorbed (Life technologies, Tokyo, Japan) and Alexa Fluor 488-conjugated donkey anti-rat IgG H&L Abs pre-adsorbed (Life technologies).

Table S1. Microarray analysis - significant upregulated genes

Symbol	Gene name/description	Genbank Accession	Fold Change
Gsdmcl-ps	Adult male testis cDNA, RIKEN full-length enriched library	AK016931	100.00
Gsdmc2	Gasdermin C2 (Gsdmc2)	NM_177912	100.00
Retnla	Resistin like alpha (Retnla)	NM_020509	93.30
Retnla	Resistin like alpha (Retnla)	NM_020509	93.56
Sprr2a	Small proline-rich protein 2A (Sprr2a)	NM_011468	100.00
Pla2g4c	Phospholipase A2, group IVC (cytosolic, calcium-independent) (Pla2g4c)	NM_001004762	100.00
Pla2g4c	Cell embryo 1 cell cDNA, RIKEN full-length enriched library, clone:I0C0013F18 product:weakly similar to Cytosolic phospholipase A2 gamma [Homo sapiens], full insert sequence	AK145339	100.00
Retnlb	Resistin like beta (Retnlb)	NM_023881	100.00
Duoxa2	Dual oxidase maturation factor 2 (Duoxa2)	NM_025777	100.00
Ccl8	Chemokine (C-C motif) ligand 8 (Ccl8)	NM_021443	47.91
Defb1	Defensin beta 1 (Defb1)	NM_007843	46.27
Defb1	Defensin beta 1 (Defb1)	NM_007843	43.24
Tpsab1	Tryptase alpha/beta 1 (Tpsab1)	NM_031187	30.24
A_51_P226791	Unknown		40.26
Ear11	Eosinophil-associated, ribonuclease A family, member 11 (Ear11)	NM_053113	42.53
St3gal4	ST3 beta-galactoside alpha-2,3-sialyltransferase 4 (St3gal4)	NM_009178	50.32
Ear6	Eosinophil-associated, ribonuclease A family, member 6 (Ear6)	NM_053111	34.43
Sprr2e	Small proline-rich protein 2E (Sprr2e)	NM_011471	37.49
Il6	Interleukin 6 (Il6)	NM_031168	47.57
Ccl1	Chemokine (C-C motif) ligand 1 (Ccl1)	NM_011329	41.66
AK088994	2 days neonate thymus thymic cells cDNA, RIKEN full-length enriched library, clone:E430034M04 product:unclassifiable, full insert sequence	AK088994	45.86
Atp13a4	ATPase type 13A4 (Atp13a4)	NM_172613	15.50
Cyp11a1	Cytochrome P450, family 11, subfamily a, polypeptide 1 (Cyp11a1), nuclear gene encoding mitochondrial protein	NM_019779	75.19
Ms4a2	Membrane-spanning 4-domains, subfamily A, member 2 (Ms4a2), mRNA	NM_013516	68.02
Cma2	Chymase 2, mast cell (Cma2)	NM_010779	69.30
Fcer1a	Fc receptor, IgE, high affinity I, alpha polypeptide (Fcer1a)	NM_010184	46.80
Gp1ba	Glycoprotein 1b, alpha polypeptide (Gp1ba)	NM_010326	74.26
Mcpt2	Mast cell protease 2 (Mcpt2)	NM_008571	98.50
Mcpt1	Mast cell protease 1 (Mcpt1)	NM_008570	92.52
Cpa3	Carboxypeptidase A3, mast cell (Cpa3)	NM_007753	73.56

Mcpt4	Mast cell protease 4 (Mcpt4)	NM_010779	81.99
Mcpt9	Mast cell protease 9 (Mcpt9)	NM_010782	78.25
Cyp11a1	Cytochrome P450, family 11, subfamily a, polypeptide 1 (Cyp11a1), nuclear gene encoding mitochondrial protein	NM_019779	70.00
Ccdc129	Coiled-coil domain containing 129 (Ccdc129)	AK085190	30.60
Gsdmc1	Gasdermin C1 (Gsdmc1)	NM_031378	100.00
Gsdmc3	Gasdermin C3 (Gsdmc3)	NM_183194	100.00
Cma1	Chymase 1, mast cell (Cma1)	NM_010780	97.46
Cma1	Chymase 1, mast cell (Cma1)	NM_010780	95.63
E130202H07Rik	Mus musculus 0 day neonate eyeball cDNA, RIKEN full-length enriched library, clone:E130202H07 product:unclassifiable, full insert sequence	AK053684	98.82
Chi3l4	Chitinase 3-like 4 (Chi3l4)	NM_145126	88.60
Cst9	Cystatin 9 (Cst9)	NM_009979	97.84
Chi3l3	Chitinase 3-like 3 (Chi3l3)	NM_009892	89.13

Table S2. Selected significant upregulated genes (Genearray analysis)

Symbol	Gene name/description	Genbank Accession	Fold Change
Ccl8	Chemokine (C-C motif) ligand 8 (Ccl8)	NM_021443	47.91
Il6	Interleukin 6 (Il6)	NM_031168	47.57
Ccl1	Chemokine (C-C motif) ligand 1 (Ccl1)	NM_011329	41.66
Il4	Interleukin 4 (Il4)	NM_021283	7.17
Il17b	Interleukin 17B (Il17b)	NM_019508	6.16
Ccr8	Chemokine (C-C motif) receptor 8 (Ccr8)	NM_007720	5.93
Il9	Interleukin 9 (Il9)	NM_008373	5.14

Table S3. ELISA antibodies

Name	Clone	Catalog	Company	Location
Purified Anti-mouse IgE	R35-72	553413	BD biosciences	Heidelberg, Germany
Biotin Rat Anti-mouse IgE	R35-118	553419	BD biosciences	Heidelberg, Germany
Anti-human/mouse IL-5 purified	TRFK5	14-7052-85	Thermo Fisher Scientific	Darmstadt, Germany
Anti-mouse IL-5 biotin	TRFK4	13-7051-85	Thermo Fisher Scientific	Darmstadt, Germany
Anti-mouse IL-4 purified	11B11	14-7041-85	Thermo Fisher Scientific	Darmstadt, Germany
Anti-mouse IL-4 biotin	BVD6-24G2	13-7042-85	Thermo Fisher Scientific	Darmstadt, Germany
Goat anti-mouse IgG1 ($_{\gamma}$1) horseradisch peroxidase conjugate		A10551	Thermo Fisher Scientific	Darmstadt, Germany
HRP-Rabbit Anti-mouse IgG2a		610220	Thermo Fisher Scientific	Darmstadt, Germany
Murine Eotaxin (CCL11) Standard ABTS ELISA Development Kit		900-K68	Peprotech	Hamburg, Germany
Mouse CCL1/TCA-3 DuoSet ELISA		DY845	R&D systems	Wiesbaden, Germany
Mouse CCL8/MCP-2DuoSet ELISA		DY790	R&D systems	Wiesbaden, Germany
MCPT1 Mouse Uncoated ELISA Kit		15561167	Thermo Fisher Scientific	Darmstadt, Germany
Ready Set Go! Anti-mouse IL-13		88-7137-22	Thermo Fisher Scientific	Darmstadt, Germany
Ready Set Go! Anti-mouse IL-33		88-7333-22	Thermo Fisher Scientific	Darmstadt, Germany

Table S4. FACS antibodies

Name	Clone	Catalog	Company	Location
FITC anti-mouse CD45, Rat IgG2b, kappa	30-F11	103108	Biolegend	Fell, Germany
Anti-mouse CD16/CD32 purified	93	14-0161-85	Thermo Fisher Scientific	Darmstadt, Germany
Anti-mouse Ly6G (Gr1) PE	RB6-8C5	12-5931-82	Thermo Fisher Scientific	Darmstadt, Germany
CD170 (Siglec F) Rat anti-Mouse, eFluor 660	1RNM44N	50-1702-82	Thermo Fisher Scientific	Darmstadt, Germany
Anti-mouse CD11b PE-Cyanine5	M1/70	15-0112-82	Thermo Fisher Scientific	Darmstadt, Germany
Anti-mouse CD4 PE	GK1.5	12-0041-83	Thermo Fisher Scientific	Darmstadt, Germany
Anti-mouse CD25 APC	PC61.5	17-0251-81	Thermo Fisher Scientific	Darmstadt, Germany
Anti-mouse/rat FoxP3 PE-Cyanine5	FJK-16s	15-5773-82	Thermo Fisher Scientific	Darmstadt, Germany

Table S5. Assessment of inflammation levels in WT and CCR8KO mice.

Group	Inflammation	Villi	Edema	Total
WT (OVA/EW)	3.75**(1),*(2)± 0.5	0.75 ± 0.5	0.75 ± 0.5	5.25**(1),*(2)± 1.5
WT (NC/EW)	0.00 ± 0.0	0.00 ± 0.0	0.00 ± 0.0	0.00 ± 0.0
CCR8KO (OVA/EW)	2.75**(3) ± 0.5	0.75 ± 0.5	0.50 ± 0.6	4.00**(3) ± 0.8
CCR8KO (NC/EW)	0.00 ± 0.0	0.00 ± 0.0	0.00 ± 0.0	0.00 ± 0.0

WT and CCR8KO mice were i.p. sensitized with OVA plus ALUM, or treated only with PBS, and fed EW-diet for 7 days. The jejunums were harvested from the mice, and stained with H&E. Inflammation levels in the tissues were assessed as follows: Inflammation grade (I) 0 rare granulocytes, isolated cells detected in the mucosa of villi and between the crypts; 1: few granulocytes scattered in the mucosa; 2: a solitary group of less than 10 granulocytes; 3: 2-3 groups up to 10 granulocytes; 4: more than 3 groups of granulocytes and larger than 10 cells. Villi (V) 0: normal, 1: shorter villi – diffused and homogeneous mild atrophy of the villi due to intestinal dilation with obvious central lumen. Edema (E) 0: not present; 1: diffused edema in the lamina propria. The total histological score represents the sum of the inflammation grade, villi and edema score. Score ranges from 0 to 6 (total score = I + V + E). OVA/EW; OVA-sensitized and EW-diet fed, NC/EW; non-sensitized and EW-diet fed. * $p<0.05$, ** $p<0.01$. (1) *vs* WT (NC/EW); (2) *vs* CCR8KO (OVA/EW); (3) *vs* CCR8KO (NC/EW).

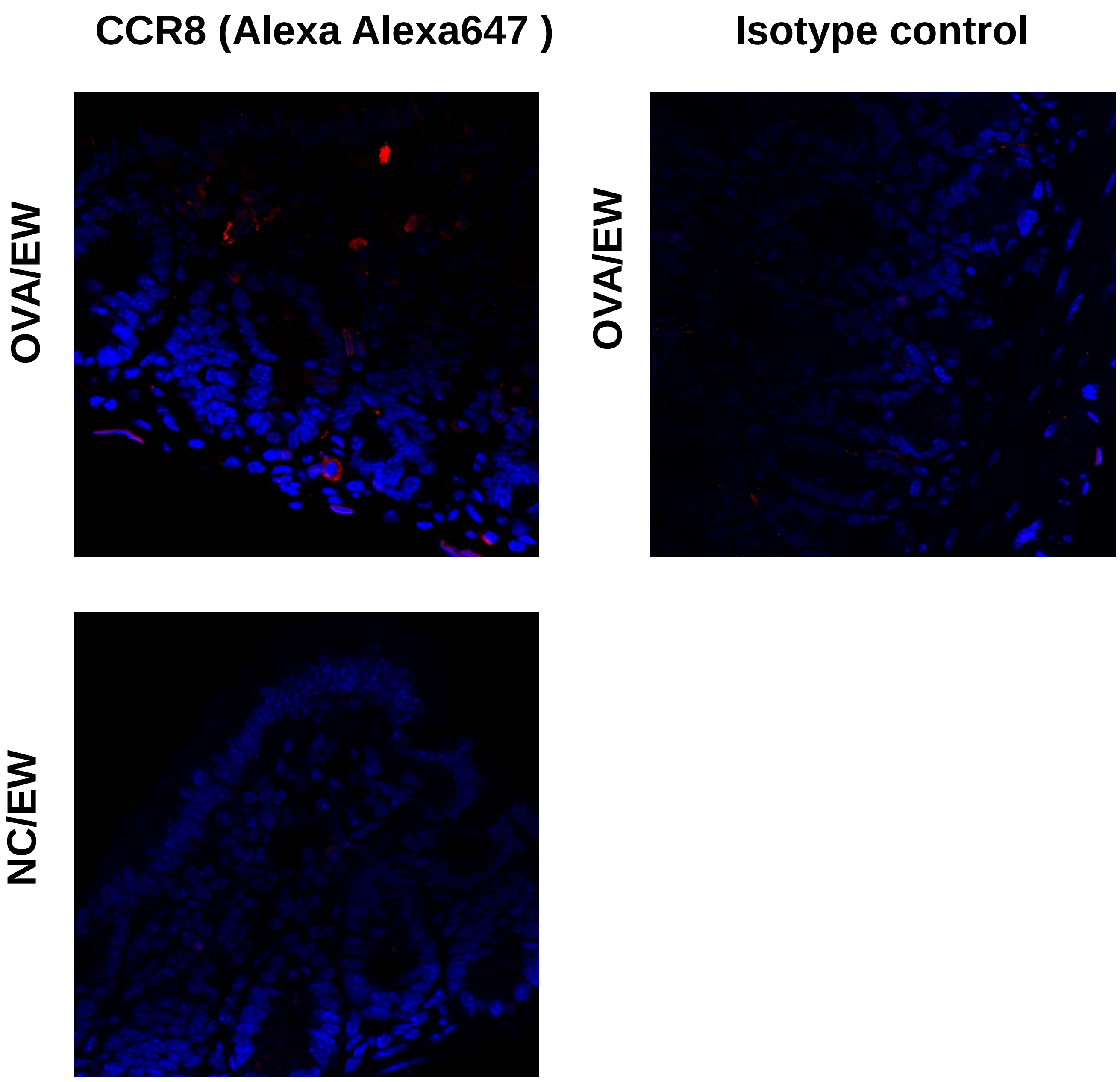

Fig. S1

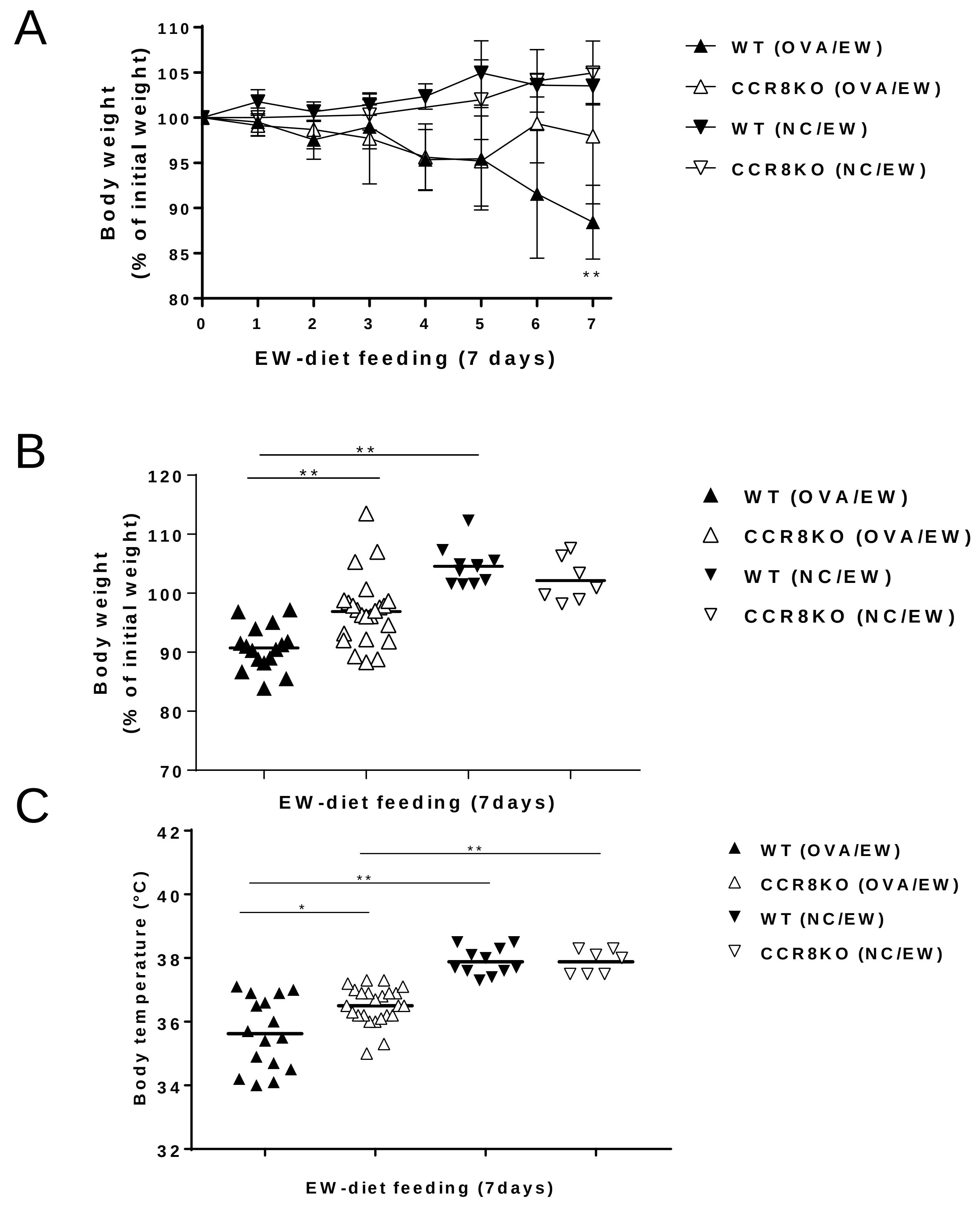
A
Body weight (% of initial weight)
EW-diet feeding (7 days)
WT (OVA/EW)
CCR8KO (OVA/EW)
WT (NC/EW)
CCR8KO (NC/EW)
**
B
Body weight (% of initial weight)
EW-diet feeding (7days)
WT (OVA/EW)
CCR8KO (OVA/EW)
WT (NC/EW)
CCR8KO (NC/EW)
**
**
C
Body temperature (°C)
EW-diet feeding (7days)
WT (OVA/EW)
CCR8KO (OVA/EW)
WT (NC/EW)
CCR8KO (NC/EW)
*
**
**

Fig. S2

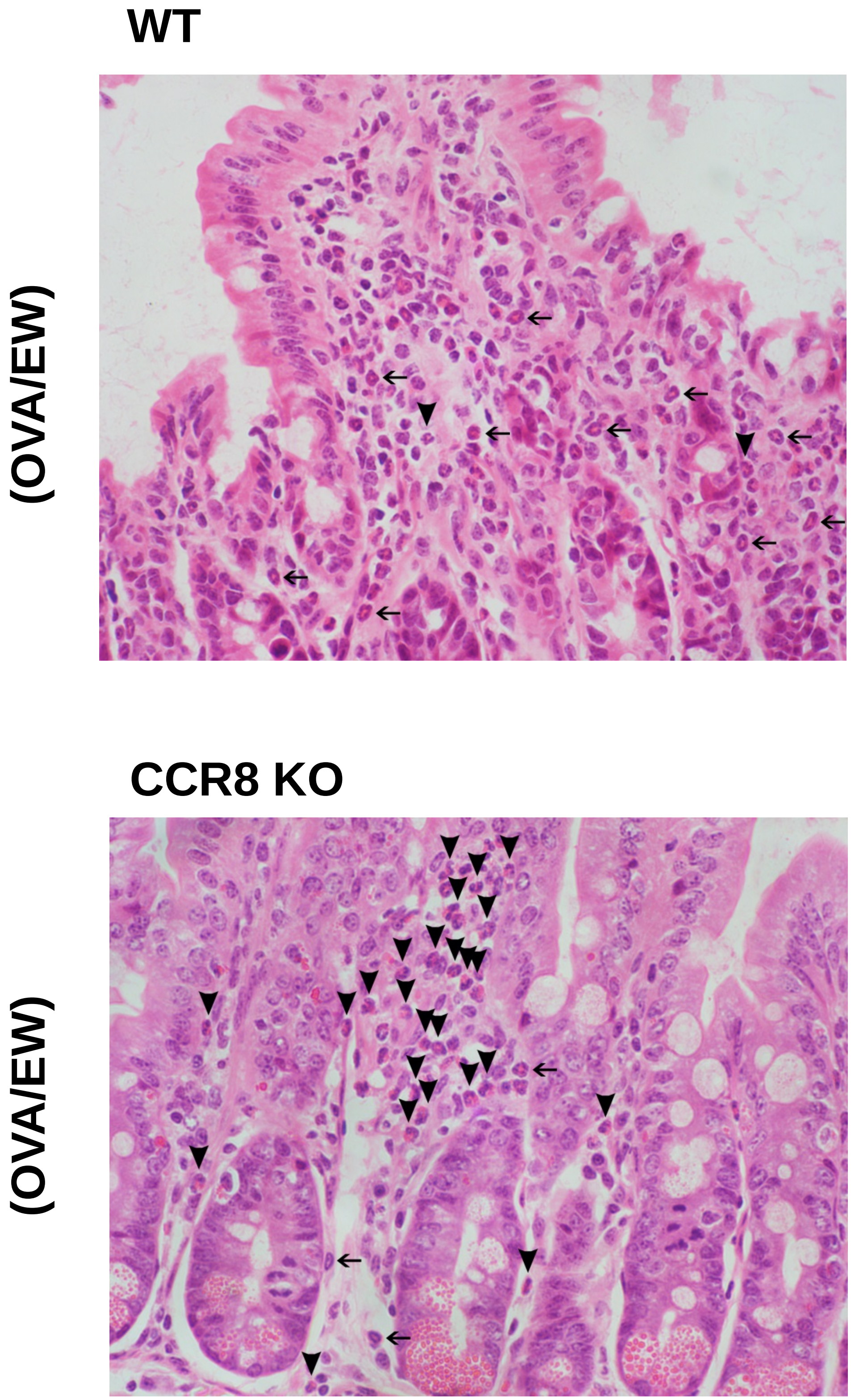

Fig. S3

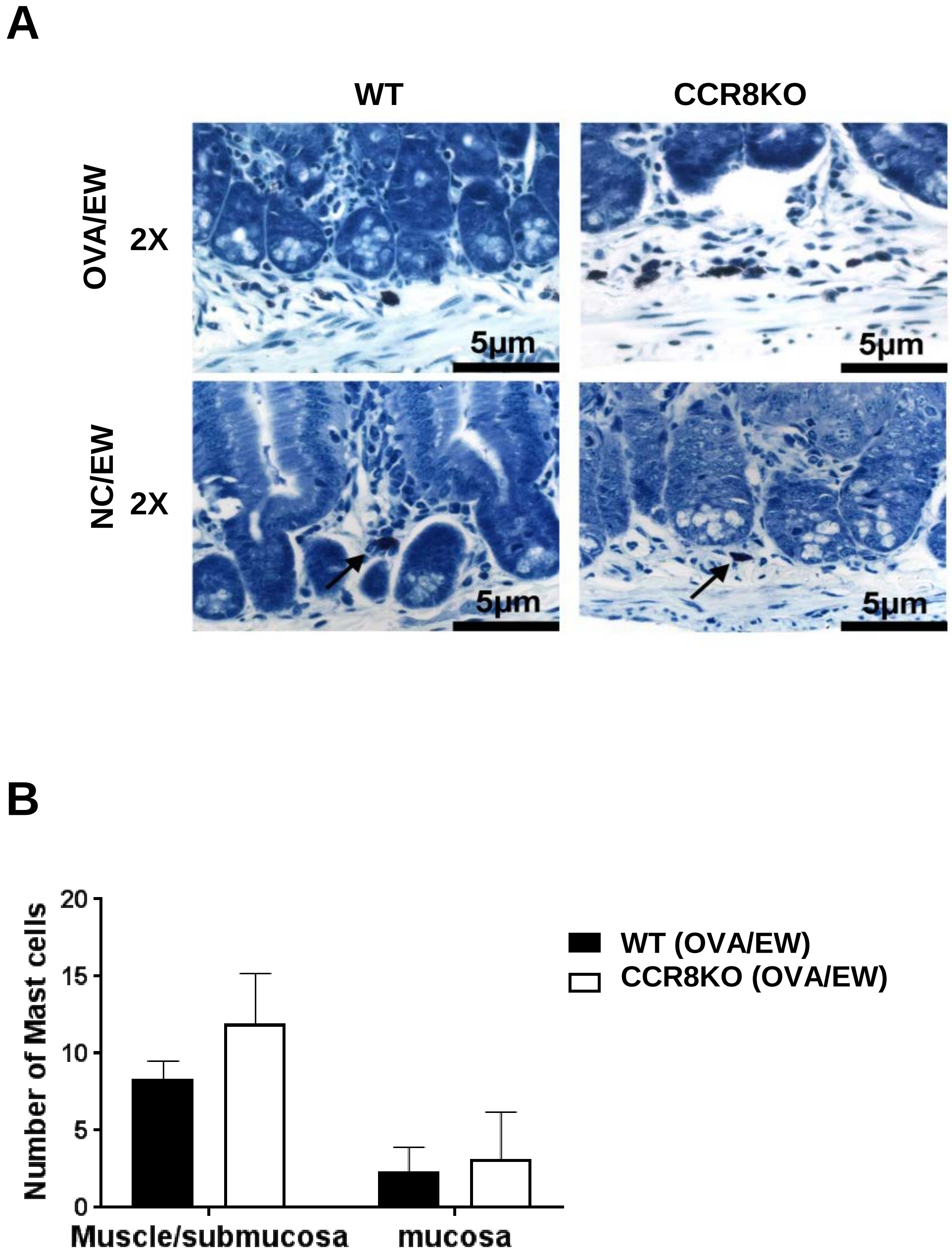
A
WT
CCR8KO
OVA/EW
2X
NC/EW
2X
5μm
5μm
5μm
5μm
B
Number of Mast cells
20
15
10
5
0
Muscle/submucosa
mucosa
WT (OVA/EW)
CCR8KO (OVA/EW)

Fig. S4

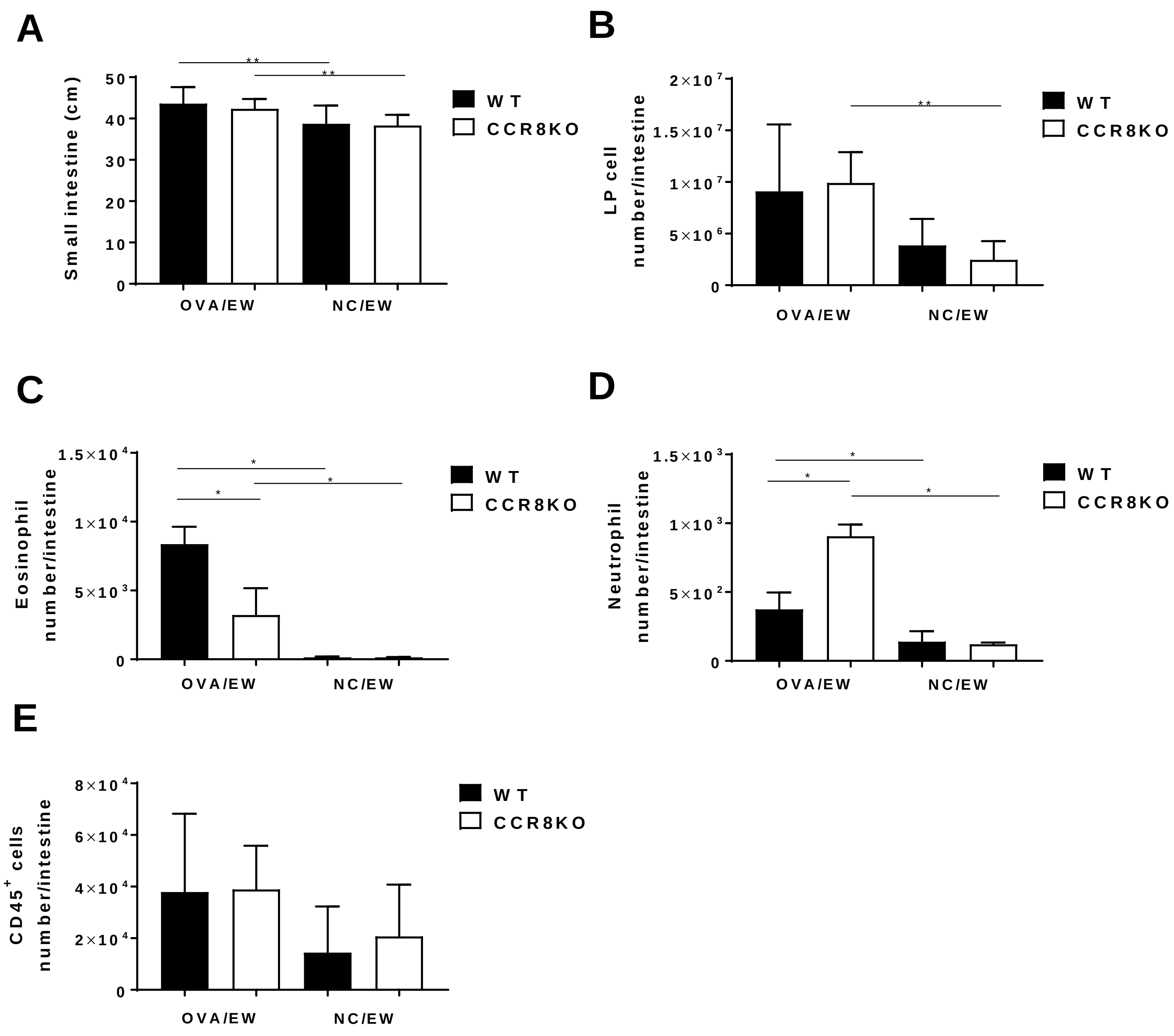
A
Small intestine (cm)
50
40
30
20
10
0
**
**
WT
CCR8KO
OVA/EW
NC/EW
B
LP cell number/intestine
2×10^7
1.5×10^7
1×10^7
5×10^6
0
**
WT
CCR8KO
OVA/EW
NC/EW
C
Eosinophil number/intestine
1.5×10^4
1×10^4
5×10^3
0
*
*
*
WT
CCR8KO
OVA/EW
NC/EW
D
Neutrophil number/intestine
1.5×10^3
1×10^3
5×10^2
0
*
*
*
WT
CCR8KO
OVA/EW
NC/EW
E
CD45+ cells number/intestine
8×10^4
6×10^4
4×10^4
2×10^4
0
WT
CCR8KO
OVA/EW
NC/EW

Fig. S5

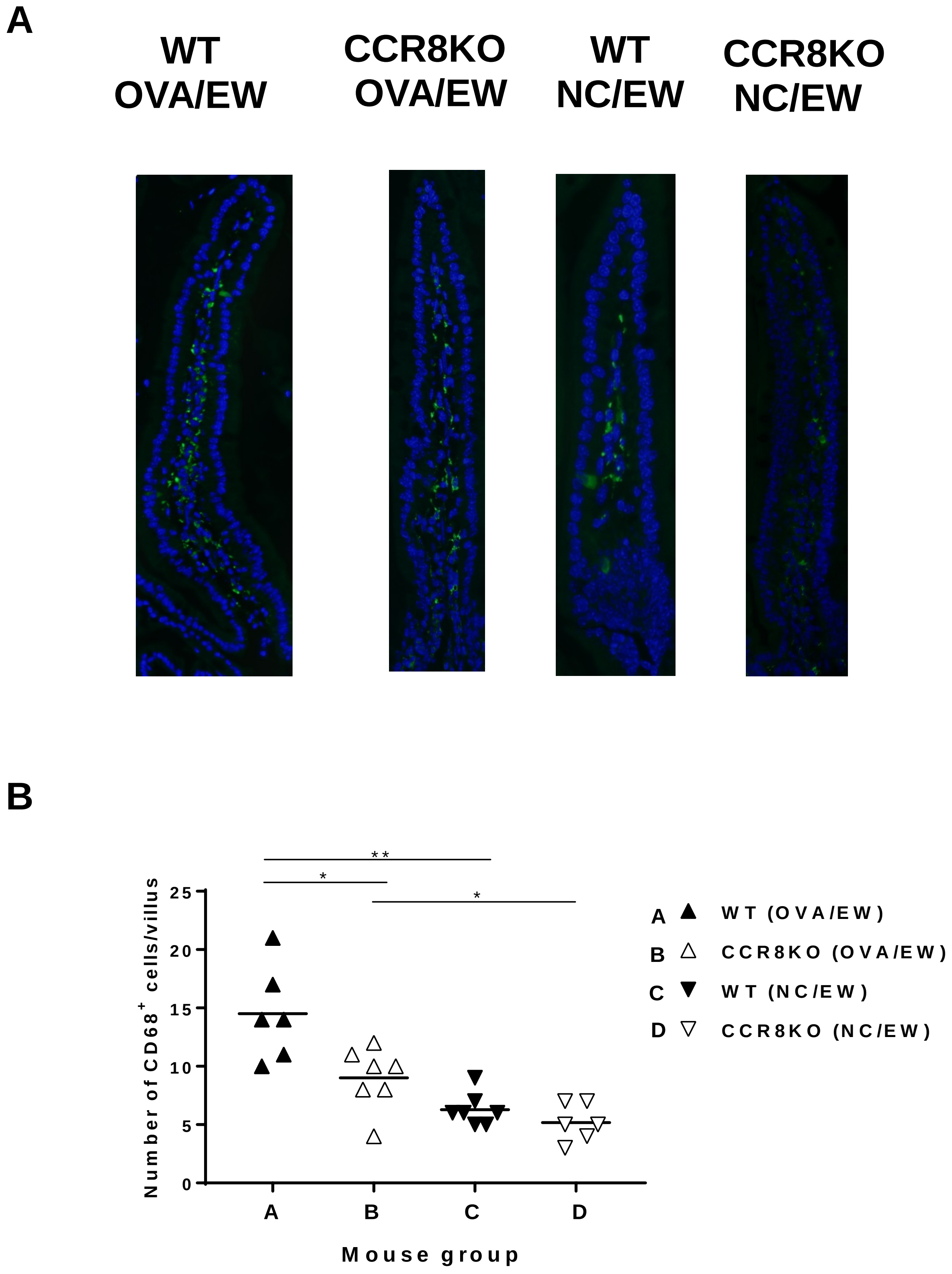
A
WT
OVA/EW
CCR8KO
OVA/EW
WT
NC/EW
CCR8KO
NC/EW
B
**
*
*
Number of CD68+ cells/villus
25
20
15
10
5
0
A
B
C
D
Mouse group
A WT (OVA/EW)
B CCR8KO (OVA/EW)
C WT (NC/EW)
D CCR8KO (NC/EW)

Fig. S6

CCR8 (Alexa Alexa647)
CD68 (Alexa 488)

Isotype controls

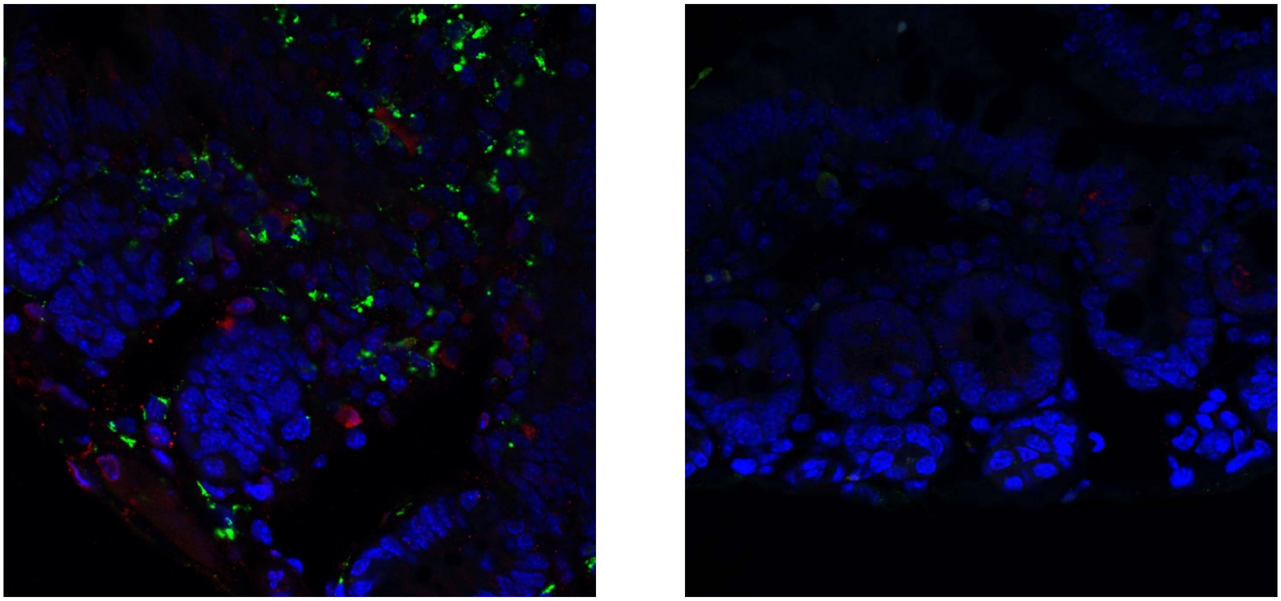

Fig. S7

Figure Legends

Fig. S1: CCR8 expression in the inflamed intestinal tissues of WT mice. WT mice were i.p. sensitized with OVA plus ALUM, and fed EW diet for 7 days. As controls, mice fed EW-diet without sensitization. The jejunums were harvested, and treated with anti-CCR8-Abs (red) or its isotype control plus propidium Iodide (blue). OVA/EW; OVA-sensitized and EW-diet fed. NC/EW; Non-sensitized and EW-diet fed. The data are representative for two independent experiments.

Fig. S2: Reduced development of clinical symptoms in CCR8KO mice. WT and CCR8KO mice were i.p. sensitized with OVA plus ALUM and fed EW-diet for 7 days. As controls, mice fed EW-diet without sensitization. (A) During EW-diet feeding, weight of the mice was monitored. The data are shown as mean ± standard deviation for each group (bars), and are representative for three independent experiments. ** $p<0.01$ vs NC/EW WT mice. (B) Weight loss of individual mice on day 7 of EW diet was indicated as a symbol. (C) On day 5 of EW diet, body temperature of the mice was measured. Each symbol represents an individual mouse. The data are pooled of three independent experiments. *$p<0.05$, **$p<0.01$. OVA/EW; OVA-sensitized and EW-diet fed. NC/EW; Non-sensitized and EW-diet fed.

Fig. S3: Neutrophil accumulation in the inflamed intestinal tissues of CCR8KO mice. WT and CCR8KO mice were i.e. sensitized with OVA plus ALUM, and fed EW-diet for 7 days. The jejunums were harvested from the mice, and stained with H&E. Eosinophils and neutrophils in their inflamed tissues mice are indicated by thin and thick arrows respectively. OVA/EW; OVA-sensitized and EW-diet fed.

Fig. S4: Comparable number of mast cells in the inflamed intestinal tissues of WT and CCR8 KO mice. WT mice and CCR8 KO mice (n=3) were i.p. sensitized with OVA plus ALUM, and fed EW diet for 7 days. The jejunums were harvested from the mice, fixed in 4% paraformaldehyde, and embedded in paraffin. The tissues were cut and stained with toluidine blue. The numbers of stained mast cells in the tissues were counted under microscope. (A) Mast cells in jejunum. (B) Number of mast cells in muscle-submucosa and mucosa. The data are shown as the average ± SD for each group (bars), and are representative of two independent experiments.

Fig. S5: Reduced eosinophil accumulation and increased neutrophil accumulation in AE tissues of CCR8KO mice. WT and CCR8KO mice were i.p. sensitized with OVA plus ALUM and fed EW-diet for 7 days. As controls, mice fed EW-diet without sensitization. On day 7 of EW diet, small intestines were harvested from the mice. (A) The length of small intestines from the mice was measured. (B) The numbers of lamina propria cells isolated from the small intestines

were counted. (C) The numbers of eosinophils and (D) the number of neutrophils in the small intestine~~s~~ were estimated, based on the data of Fig. 5B and Fig. S5A. (E) The number of CD45 positive cells in the laminar propria cells was estimated. OVA/EW; OVA-sensitized and EW-diet fed. NC/EW; Non-sensitized and EW-diet fed. The data are shown as mean ± standard deviation for each group (bars), and are representative for three independent experiments. *$p<0.05$, **$p<0.01$.

Fig. S6: Reduced number of CD68 positive cells in AE tissues of CCR8 KO mice. WT mice and CCR8KO mice were i.p. sensitized with OVA plus ALUM and fed EW-diet for 7 days. As controls, mice fed EW-diet without sensitization. On day 7 of EW diet, jejunums were harvested from the mice. (A) The tissues were stained with anti-CD68 mAb (green) and propidium Iodide (blue). (B) The averaged numbers of CD68 positive cells/villus in the jejunum of the mice were counted. OVA/EW; OVA-sensitized and EW-diet fed. NC/EW; Non-sensitized and EW-diet fed. * $P<0.05$.

Fig. S7: CD68 and CCR8 expression in the inflamed tissues of WT mice. WT mice were i.p. sensitized with OVA plus ALUM and fed EW-diet for 7 days. On day 7 of EW diet, jejunums were harvested from the mice. The tissues were stained with anti-CD68 mAb (green), anti-CCR8-Abs (red), or isotype controls plus propidium Iodide (blue).
